# Leptin and Pro-Inflammatory Stimuli Synergistically Upregulate MMP-1 and MMP-3 Secretion in Human Gingival Fibroblasts

**DOI:** 10.1371/journal.pone.0148024

**Published:** 2016-02-01

**Authors:** Rachel C. Williams, Andrew J. Skelton, Stephen M. Todryk, Andrew D. Rowan, Philip M. Preshaw, John J. Taylor

**Affiliations:** 1 Centre for Oral Health Research, Newcastle University, Newcastle upon Tyne, United Kingdom; 2 Musculoskeletal Research Group, Newcastle University, Newcastle upon Tyne, United Kingdom; 3 Bioinformatics Support Unit, Newcastle University, Newcastle upon Tyne, United Kingdom; 4 Institute of Cellular Medicine, Newcastle University, Newcastle upon Tyne, United Kingdom; 5 Department of Applied Sciences, Faculty of Health and Life Sciences, Northumbria University, Newcastle upon Tyne, United Kingdom; INRS, CANADA

## Abstract

**Introduction:**

Gingival fibroblast-mediated extracellular matrix remodelling is implicated in the pathogenesis of periodontitis, yet the stimuli that regulate this response are not fully understood. The immunoregulatory adipokine leptin is detectable in the gingiva, human gingival fibroblasts express functional leptin receptor mRNA and leptin is known to regulate extracellular matrix remodelling responses in cardiac fibroblasts. We therefore hypothesised that leptin would enhance matrix metalloproteinase secretion in human gingival fibroblasts.

**Methods and Results:**

We used in vitro cell culture to investigate leptin signalling and the effect of leptin on mRNA and protein expression in human gingival fibroblasts. We confirmed human gingival fibroblasts expressed cell surface leptin receptor, found leptin increased matrix metalloproteinase-1, -3, -8 and -14 expression in human gingival fibroblasts compared to unstimulated cells, and observed that leptin stimulation activated MAPK, STAT1/3 and Akt signalling in human gingival fibroblasts. Furthermore, leptin synergised with IL-1 or the TLR2 agonist pam2CSK4 to markedly enhance matrix metalloproteinase-1 and -3 production by human gingival fibroblasts. Signalling pathway inhibition demonstrated ERK was required for leptin-stimulated matrix metalloproteinase-1 expression in human gingival fibroblasts; whilst ERK, JNK, p38 and STAT3 were required for leptin+IL-1- and leptin+pam2CSK4-induced matrix metalloproteinase-1 expression. A genome-wide expression array and gene ontology analysis confirmed genes differentially expressed in leptin+IL-1-stimulated human gingival fibroblasts (compared to unstimulated cells) were enriched for extracellular matrix organisation and disassembly, and revealed that matrix metalloproteinase-8 and -12 were also synergistically upregulated by leptin+IL-1 in human gingival fibroblasts.

**Conclusions:**

We conclude that leptin selectively enhances the expression and secretion of certain matrix metalloproteinases in human gingival fibroblasts, and suggest that gingival fibroblasts may have an ECM-degrading phenotype during conditions of hyperleptinaemia (e.g., obesity, type 2 diabetes mellitus, exogenous leptin therapy).

## Introduction

Gingival connective tissue is predominantly composed of stromal cells, such as fibroblasts, and a collagen-rich extracellular matrix (ECM). Fibroblasts control the formation and remodelling of the gingival ECM, through regulated expression of ECM components and ECM-remodelling enzymes such as matrix metalloproteinases (MMPs) [1]. Fibrillar collagens are degraded by the collagenolytic MMPs (MMP-1, MMP-8 and MMP-13) [2]. Excessive destruction of ECM proteins, such as collagen, can be irreversible and is a feature of the common, chronic inflammatory disease periodontitis. Therefore, ECM remodelling is tightly regulated [3]. Exogenous pro-inflammatory stimuli such as bacterial lipopolysaccharide (LPS) are implicated in the pathogenesis of periodontitis [4] and up-regulate the expression of several MMPs in human gingival fibroblasts (HGFs) [5]. Additionally, the IL-6 family cytokine oncostatin-M (OSM), and IL-1 synergistically increase the secretion of MMP-1 by human gingival fibroblasts (HGFs) [6].

Obesity and type 2 diabetes mellitus are both positively associated with periodontitis [7], however the molecular mechanisms underpinning this relationship are poorly characterised [8]. The profile of circulating cytokines, growth factors and adipokines is altered in obese individuals and those with type 2 diabetes [9], and HGFs respond to many of these molecules. For example, the adipokine adiponectin partially suppresses IL-1-stimulated IL-6 and IL-8 production by HGFs [10]. Changes in the regulation, interactions and functionality of these mediators may link obesity, diabetes and periodontitis [8]. Circulating levels of the adipokine leptin are proportional to total adipose tissue mass, and the primary functions of leptin are to inform the brain of energy reserves and to regulate energy expenditure [11]. However, leptin also plays roles in angiogenesis, fertility, bone metabolism, immunity, wound repair and haematopoiesis, and is classified as a member of the IL-6 cytokine family [12,13]. Leptin has been detected in the gingiva by immunohistochemistry [14] and HGFs express functional leptin receptor (LEPR) mRNA [15]. Functionally, leptin increases the secretion of IL-6 and IL-8 by HGFs [15]. In this study we examined whether leptin, either alone or in combination with inflammatory mediators, could alter the expression of genes involved in ECM remodelling in HGFs, with a focus on the collagenase MMP-1, and the stromelysin MMP-3. We found that leptin enhanced the secretion of MMP-1 and MMP-3, and in combination with IL-1 synergistically up-regulated MMP-1, MMP-3, MMP-8 and MMP-12 expression by HGFs. Mechanistically, we found that leptin+IL-1-induced MMP-1 expression in HGFs was regulated by MAPK and STAT3 signalling.

## Materials and Methods

### Reagents and Antibodies

Recombinant human (rh) leptin was from Biotechne (Abingdon, UK). In most experiments rhIL-1α was used as an IL-1 receptor agonist as described previously (Catterall, 2001); rhIL-1β was a gift from Dr Keith Ray (GlaxoSmithKline, Stevenage, UK). In microarray experiments IL-1β (Biotechne) was used. RhOSM was prepared as described previously [16]. Pam2CSK4 and LPS from *E*. *coli* strain 0111:B4 were from Invivogen (Source Bioscience, Nottingham, UK). Inhibitors of JNK (SP600125) and ERK (U0126) were from Biotechne, inhibitors of p38 (SB203580), STAT3 (S3I-201) and Akt (inhibitor VIII) were from Merck Millipore (Watford, UK). Monoclonal antibodies (Abs) against phospho-STAT1 (Y701), phospho-STAT3 (Y705), STAT-3, phospho-p38 (T180/Y182), phospho-JNK (T183/Y185), phospho-ERK1/2 (T202/Y204), ERK1/2 and phospho-Akt (S473) were from Cell-Signalling Technologies (New England Biolabs, Hitchin, UK), GAPDH Ab from Merck-Millipore, HRP-conjugated anti-mouse and anti-rabbit Ig Abs from Dako (Ely, UK), IgG2B mouse LEPR and isotype control PE-conjugated Abs from R&D Systems, IgG2A mouse Toll-like receptor (TLR)2 and TLR4 APC-conjugated Abs were from EBioscience (Hatfield, UK), and an isotype control APC-conjugated Ab was from AbD Serotec (Kidlington, UK). Except where indicated, all other reagents were supplied by Sigma-Aldrich (Gillingham, UK).

### HGF isolation and culture

**Fig 1:**
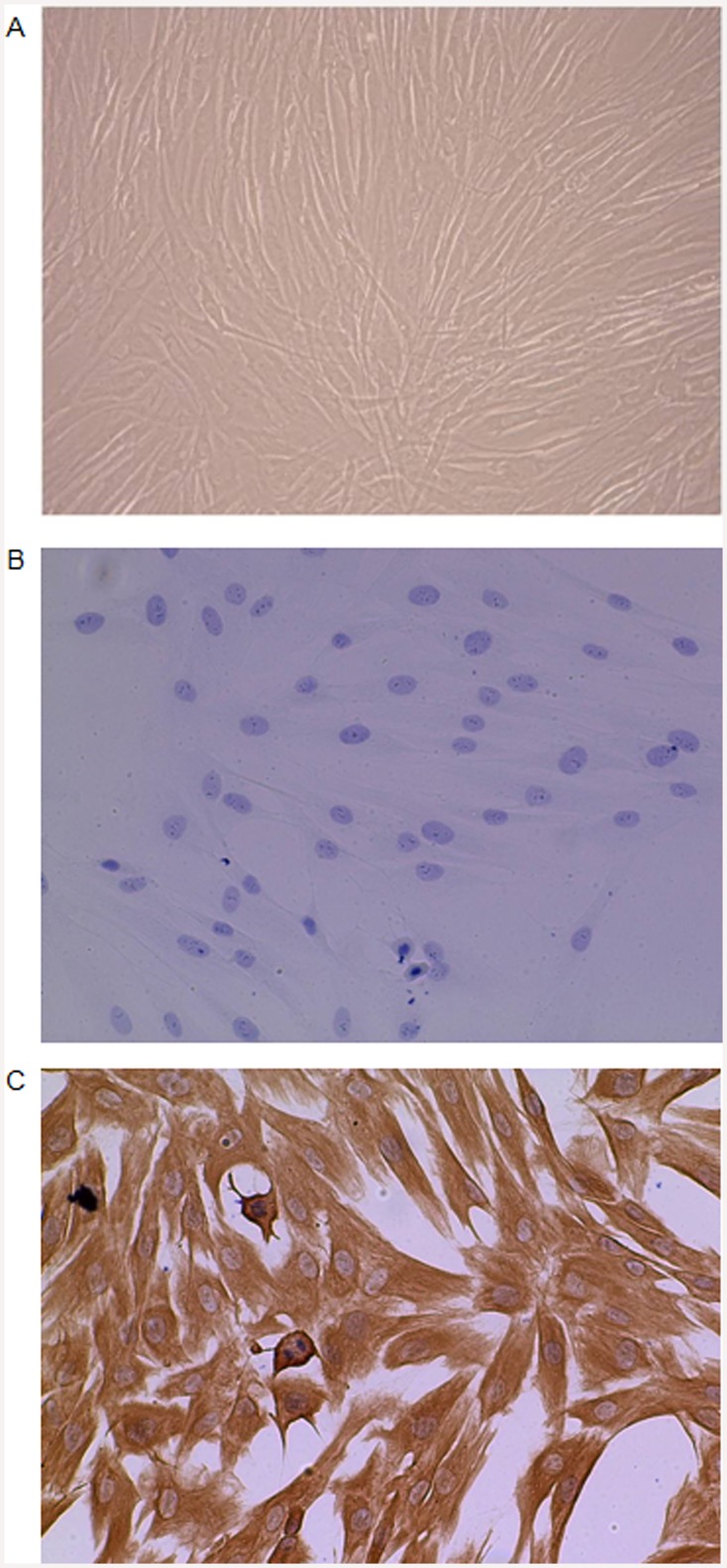
HGF morphology and vimentin expression. (A) Light microscopy (100x magnification) of viable HGFs at high confluency displaying characteristic fibroblast morphology. HGFs stained with (B) no antibody control (C) anti-vimentin Ab. Images presented are from one donor and are representative of similar micrographs form three donors stained independently.

Primary HGFs were derived from healthy gingival tissue obtained from patients with written informed consent undergoing canine tooth exposure surgery, with ethical approval from the National Research Ethics Service Committee North East (Reference: 07/Q1003/41). The tissue was collected on ice into F-12 Ham’s medium, washed in PBS (both supplemented with 200 U/ml penicillin, 200 μg/ml streptomycin and 80 U/ml nystatin), dissected free of gingival epithelium and cut into 2 mm^3^ sections. Sections were incubated (5% CO_2_ at 37°C) in DMEM (containing 4.5 g/l glucose and supplemented with 100 U/ml penicillin, 100 μg/ml streptomycin, 2 mM L-glutamine and 10% FBS) to allow HGF outgrowth. HGFs were maintained in DMEM as previously described [17] and examined for morphology and expression of the mesenchymal marker vimentin (Fig 1). Briefly, HGFs were fixed in methanol, then washed in TBS (pH 7.6), prior to incubation with mouse monoclonal anti human vimentin Ab (IgG2b, Clone Vim 3B4) (Dako) or diluent alone (3% FBS/TBS). After washing in TBS, HGFs were incubated in Envision polymer (Dako) for 30 min. HGFs were washed again in TBS before incubation in diaminobenzidine solution (Dako) for 5 min, then counterstained with haematoxylin. Excess diaminobenzidine and haematoxylin were removed by washing in water. HGFs between passages 5 and 9 were serum-starved for 18 h before a 30 min pre-treatment with chemical pathway inhibitors or DMSO (<0.1%), followed by stimulation with leptin (0.1–25 μg/ml), IL-1 (0.05 ng/ml), OSM (5 ng/ml), pam2CSK4 (50 ng/ml) and/or LPS (10 ng/ml) for 24 h as described previously [18–20]. Inhibitors and DMSO were used at concentrations that were empirically demonstrated to have no adverse effect on HGF viability at 24 h (not shown).

### RNA extraction and RT-PCR

RNA was extracted using commercial kits (GenElute Mammalian Total RNA Miniprep Kit; RNeasy Mini Kit, Qiagen, Manchester, UK; SideStep Lysis and Stabilization Buffer, Agilent Technologies, Wokingham, UK) and reverse transcribed using the High-Capacity cDNA Reverse Transcription kit (Life Technologies, Paisley, UK). Oligonucleotides and PrimeTime qPCR probes (Integrated DNA Technologies, Glasgow, UK) used for real-time RT-PCR are shown in Table 1. TaqMan assays for RNA polymerase II (RNAP) (*POLR2A* Hs00172187_m1) and Collagen Type 6A3 (*COL6A3* Hs00915125_m1) were from Life Technologies. For real-time RT-PCR, relative gene expression levels were determined in cDNA samples using TaqMan Universal Master Mix (Life Technologies) and the ABI Prism 7900HT sequence detection system (Life Technologies). Expression levels were normalised to RNAP. Primers used for conventional real-time RT-PCR are shown in Table 2. For RT-PCR, cDNA was amplified using Bio-Mix Red PCR reaction mix (Bioline, London, UK) and analysed on 3.5% agarose gels stained with Gel-Red (Biotium, Cambridge Bioscience, Cambridge, UK) using β2-microglobulin (β2m) or 18S rRNA as a reference gene.

**Table 1:** Primer and probe sequences used for real-time RT-PCR.

Gene	Forward	Reverse	Probe
***MMP-1***	5′-AAGATGAAAGGTGGAC CAAAATT-3	5′-CCAAGAGAATGGCCGAG TTC-3′	5′-FAM-CAGAGAGTACAACTTACATCGTGTTGCGG CTC-TAMRA-3′
***MMP-2***	5’-TGGCGATGGATACCCC TTT-3’	5’-TTCTCCCAAGGTCCATAG CTCAT-3’	5’-FAM-CTCCTGGCTCATGCCTT CGCCC-TAMRA-3’
***MMP-3***	5’-TTCCGCCTGTCTCAAG ATGATAT-3’	5’-AAAGGACAAAGCAGGAT CACAGT T-3’	5’-FAM-TCAGTCCCTCTATGGACCT CCCCCTGAC-TAMRA-3’
***MMP-7***	5’-CTTTGCGCGAGGAGCT CA-3’	5’-CAGGCGGAAAGGCATGA-3’	5’-FAM-CCATTTGATGGGCCAGGA AACACG-TAMRA-3’
***MMP-8***	5’-CACTCCCTCAAGATGA CATCGA-3’	5’-ACGGAGTGTGGTGATAG CATCA-3’	5’-FAM-CAAGCAACCCTATCCAAC CTACTGGAC CAA-TAMRA-3’
***MMP-9***	5’-CCTGGGCAGATTCCAA ACCT-3’	5’-GCAAGTCTTCCGAGTAGT TTTGGA T-3’	5’-FAM-CTCAAGTGGCACCACCAC AACATCACC-TAMRA-3’
***MMP-10***	5’-GGACCTGGGCTTTATG GAGATAT-3’	5’-CCCAGGGAGTGGCCAA GT-3’	5’-FAM-CATCAGGCACCAATTTATC CTCGTTGCT-TAMRA-3’
***MMP-12***	5’-CGCCTCTCTGCTGATG ACATAC-3’	5’-GGTAGTGACAGCATCAA AACTCAA A-3’	5’-FAM-TCCCTGTATGGAGACCCA AAAGAGAA CCA-TAMRA-3’
***MMP-13***	5′-AAATTATGGAGGAGAT GCCCATT-3′	5′-TCCTTGGAGTGGTCAAGA CCTAA-3′	5′-FAM-CTACAACTTGTTTCTTGTT GCTGCGCATGA-TAMRA-3′
***MMP-14***	5’-AGGCCGACATCATGAT CTTCTTT-3’	5’-AAGTGGGTGTCTCCTCCA ATGTT-3’	5’-FAM-CCATGGCGACAGCACGCC CTT-TAMRA-3’
***TIMP-1***	5’-GACGGCCTTCTGCAAT TCC-3’	5’-GTATAAGGTGGTCTGGTT GACTTCTG-3’	5’-FAM-ACCTCGTCATCAGGGCCA AGTTCGT-TAMRA-3’
***TIMP-2***	5’-GAGCCTGAACCACAGG TACCA-3’	5’-AGGAGATGTAGCACGGG ATCA-3’	5’-FAM-CTGCGAGTGCAAGATCAC GCGC-TAMRA-3’
***TIMP-3***	5’- CCA GGA CGC CTT CTG CAA-3’	5’- CCC CTC CTT TAC CAG CTT CTT C-3’	

**Table 2:** Primer sequences used for conventional RT-PCR.

Gene	Forward	Reverse	Product size
***MMP-3***	5’-TTCCGCCTGTCTCAAG ATGATAT-3’	5’-AAAGGACAAAGCAGGAT CACAGT T-3’	150 bp
***18S rRNA***	5′-CGAATGGCTCATTAAA TCAGTTATG G-3′	5′-TATTAGCTCTAGAATTAC CACAGT TATCC-3	85 bp
***LEPR***	5’-GAAGATGTTCCGAACC CCAAGAATT G-3’	5’-CTAGAGAAGCACTTGGT GACTGAA C-3’	428 bp
***β2m***	5’-ACCCCCACTGAAAAAG ATGA-3’	5’-CTTATGCACGCTTAACTA TC-3’	425 bp

### ELISA, flow cytometry and immunoblotting

Culture supernatants from HGFs were analysed for total human MMP-1 and MMP-3 by ELISA (R&D systems) according to the manufacturer’s protocol. These assays measure total MMPs (i.e. both proenzymes and active forms) Expression of LEPR on the cell surface of HGFs was analyzed by flow cytometry. ≥ 10,000 gated events were acquired on a FACSCalibur flow cytometer (Becton Dickinson, Oxford, UK). Data were analyzed with WinMDI 2.8 software (Joseph Trotter, The Scripps Research Institute, Purdue University, West Lafayette, IN, USA). Western blotting was performed as described previously [18].

### Microarray transcriptional profiling

HGFs from three donors were stimulated with leptin (10 μg/ml) ± IL-1β (0.05 ng/ml) for 24 h in independent experiments and RNA was isolated using a RNeasy Mini Kit. Quality control, RNA amplification, hybridisation onto the HumanHT-12 v4 expression beadchip (Illumina, Little Chesterford, UK) and data generation were performed by Cambridge Genomic Services (Cambridge, UK).

Expression datasets were normalised using the robust spline method and principal component analysis was performed in R (Bioconductor, Seattle, WA, USA). Principle component analysis identified that the datasets from one donor were different to the others in one dimension (not shown). This variation was eliminated using ComBat/surrogate variable analysis [21,22]. The Human HT-12 v4 expression beadchip consists of > 47,000 probes and covers 31,000 genes. Differentially expressed genes for selected comparisons were determined as those having a corrected p value of <0.01 (as assessed using Limma with Benjamini-Hochberg correction for multiple comparisons) and a fold change ≥2. Gene ontology (GO) overrepresentation analysis (biological processes only) was performed using the GOstats Bioconductor library in R. The datasets used in this study are deposited in Gene Expression Omnibus (Ref: GSE68685) in accordance with Minimum Information About a Microarray Experiment (MIAME).

### Statistical analysis

Statistical analysis was performed using SPSS 15.0. Shapiro-Wilk testing for normality and Levene testing for homogeneity of variance were performed prior to post hoc analyses of parametric and non-parametric data by Student’s t test and Mann Whitney U test respectively. Real-time RT-PCR data were analysed using ΔCt values [23]. *P* values were corrected for multiple comparisons using the Bonferroni-Holm correction. A *P* value of <0.05 was considered significant.

## Results

### Leptin upregulates MMP expression in HGFs

**Fig 2:**
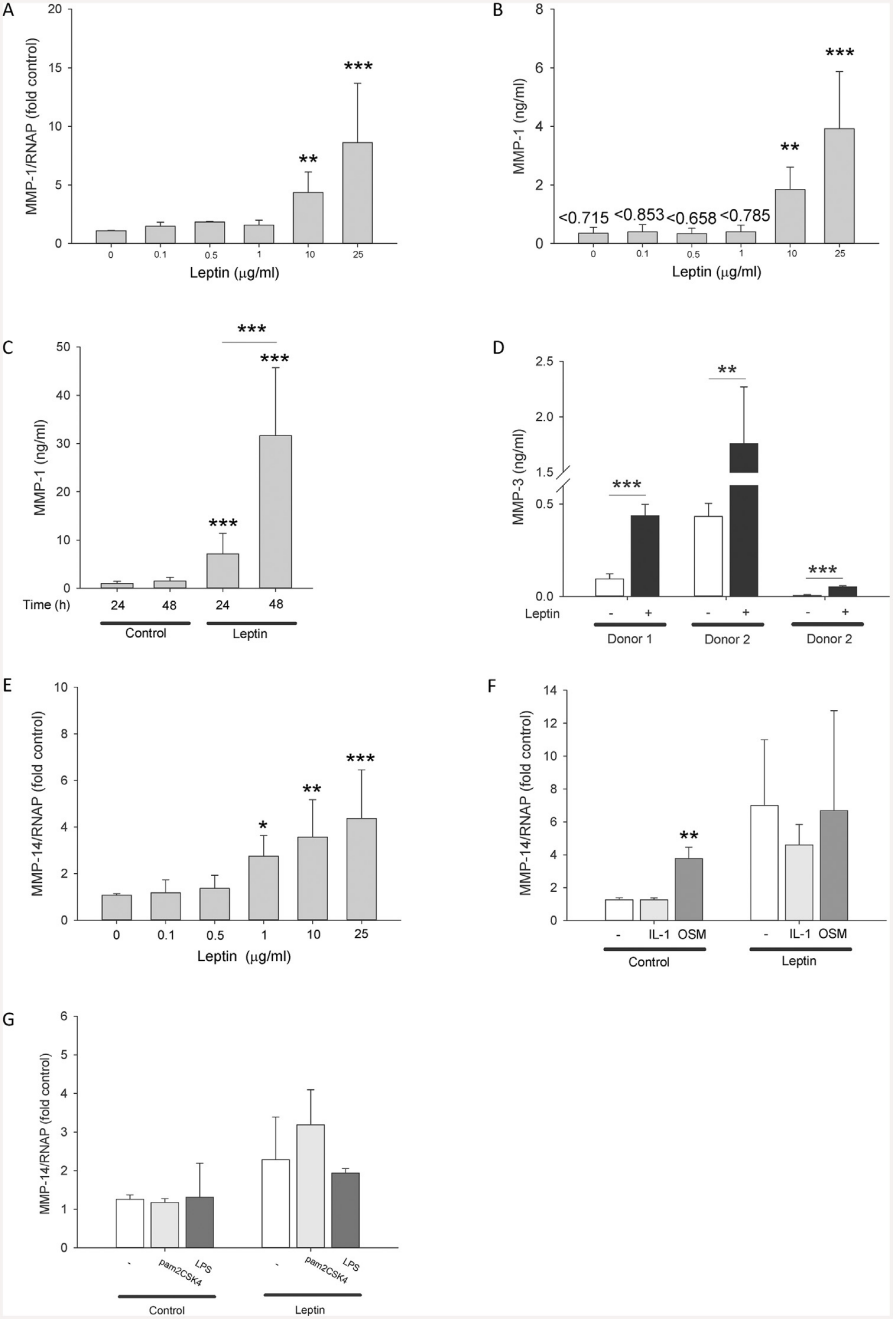
Effect of leptin on MMP mRNA and protein synthesis in HGFs. HGFs were stimulated with leptin (0–25 μg/ml) for 24 h; *MMP-1* gene expression was assessed by real-time RT-PCR (A) and supernatant MMP-1 concentrations were assessed by ELISA (B). HGFs were stimulated with leptin (10 μg/ml) for 24 h or the indicated durations and supernatant MMP-1 (C) and MMP-3 (D) concentrations were assessed by ELISA. HGFs were stimulated with leptin (0–25 μg/ml) for 24 h and *MMP-14* gene expression was assessed by real-time RT-PCR (E). HGFs were stimulated with IL-1α (0.05 ng/ml) or OSM (5 ng/ml) alone, or in combination with leptin (10 μg/ml) (F) and pam2CSK4 (50 ng/ml) or *E*. *coli* LPS (10 ng/ml) alone, or in combination with leptin (10 μg/ml) for 24 h (**G**) and *MMP-14* gene expression was determined by real-time RT-PCR. RT-PCR data (fold control) are expressed relative to RNAP. Data (excluding Fig 2D) are presented as mean+SEM HGF cultures from 3 HGF donors stimulated in independent experiments (n = 4 from each donor). Remaining data (Fig 2D) are expressed as mean+SD from 3 individual HGF donors (n = 4 from each donor). *P<0.05, **P<0.01, ***P<0.001 compared to the unstimulated control at the same time point.

Leptin increased the expression of the collagenase *MMP-1* in HGFs in a dose-dependent manner (Fig 2A; Table 3). Similar effects on MMP-1 protein secretion were also observed in HGFs (Fig 2B). This increased MMP-1 secretion was observed in a time-dependent manner (Fig 2C). The collagenase MMP-8 was also upregulated by leptin in HGFs (Table 3). In contrast, the collagenase *MMP-13* was not induced by leptin (Table 3). Leptin had no effect on the expression of the gelatinases (*MMP-2/MMP-9*), the matrilysin *MMP-7*, the metalloelastase *MMP-12* and tissue inhibitor of metalloproteinase (*TIMP*)-1-3, although most of these genes were expressed basally in HGFs (Table 3). Similarly, leptin had no effect on the expression of the stromelysins *MMP-3* and *MMP-10* in HGFs; however, the expression levels of *MMP-3* were low and not consistently detected in all donors by RT-PCR. MMP-3 protein secretion in HGFs was increased by leptin though the absolute levels of expression were donor-dependent ranging between 51.9±5.2–1760±510 pg/ml (Fig 2D). Leptin increased the expression of the membrane-type *MMP-14* in HGFs in a dose-dependent manner (Fig 2E). The related IL-6 family cytokine OSM, but not IL-1, pam2CSK4 or LPS, similarly increased *MMP-14* expression (Fig 2F and 2G).

**Table 3:** Basal and leptin-stimulated MMP and TIMP expression in HGFs.

Group	Gene	Basal expression	Effect of leptin on mRNA expression	Number of donors tested
Collagenase	*MMP-1*	+	Upregulated at ≥ 10 μg/	7
	*MMP-8*	+	Upregulated at 10 μg/ml	4
	*MMP-13*	-	No effect	4
Gelatinase	*MMP-2*	+	No effect	3
	*MMP-9*	-	No effect	3
Stromelysin	*MMP-3*	+/-	No effect	4
	*MMP-10*	+	No effect	3
Matrilysin	*MMP-7*	-	No effect	3
Membrane-type MMP	*MMP-14*	+	Upregulated at ≥ 1 μg/ml	3
Other MMPs	*MMP-12*	+	No effect	3
TIMPs	*TIMP-1*	+	No effect	4
	*TIMP-2*	+	No effect	3
	*TIMP-3*	+	No effect	3

Gene expression was determined by real-time RT-PCR. + indicates basal gene expression in all donors, +/- indicates basal gene expression in some donors, - indicates no basal gene expression in all donors assessed. ‘No effect’ indicates no effect at ≤ 25 μg/ml

### Leptin and IL-1 or pam2CSK4 synergistically upregulate MMP-1 and MMP-3 expression in HGFs

**Fig 3:**
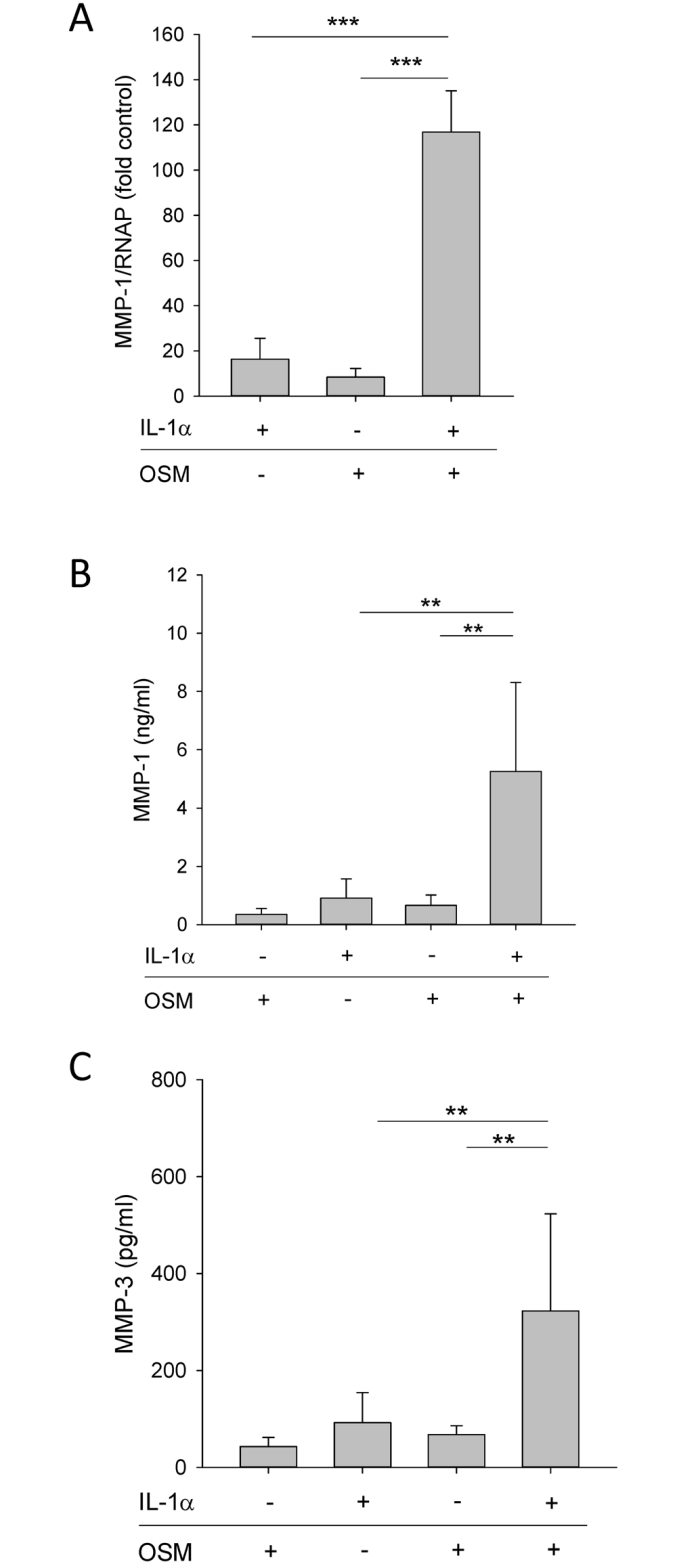
Effect of IL-1 and OSM on MMP-1 and MMP-3 production by HGFs. HGFs were stimulated with IL-1α (0.05 ng/ml), OSM (5 ng/ml) or IL-1α+OSM for 24 h. (A) *MMP-1* gene expression was assessed by real-time RT-PCR and data (fold control) are expressed relative to RNAP. Supernatant MMP-1 (B) and MMP-3 (C) concentrations were assessed by ELISA. Data are presented as mean+SEM of three donors stimulated in independent experiments (n = 4 for each donor). **P<0.01, ***P<0.001 compared to the unstimulated control at the same time point.

**Fig 4:**
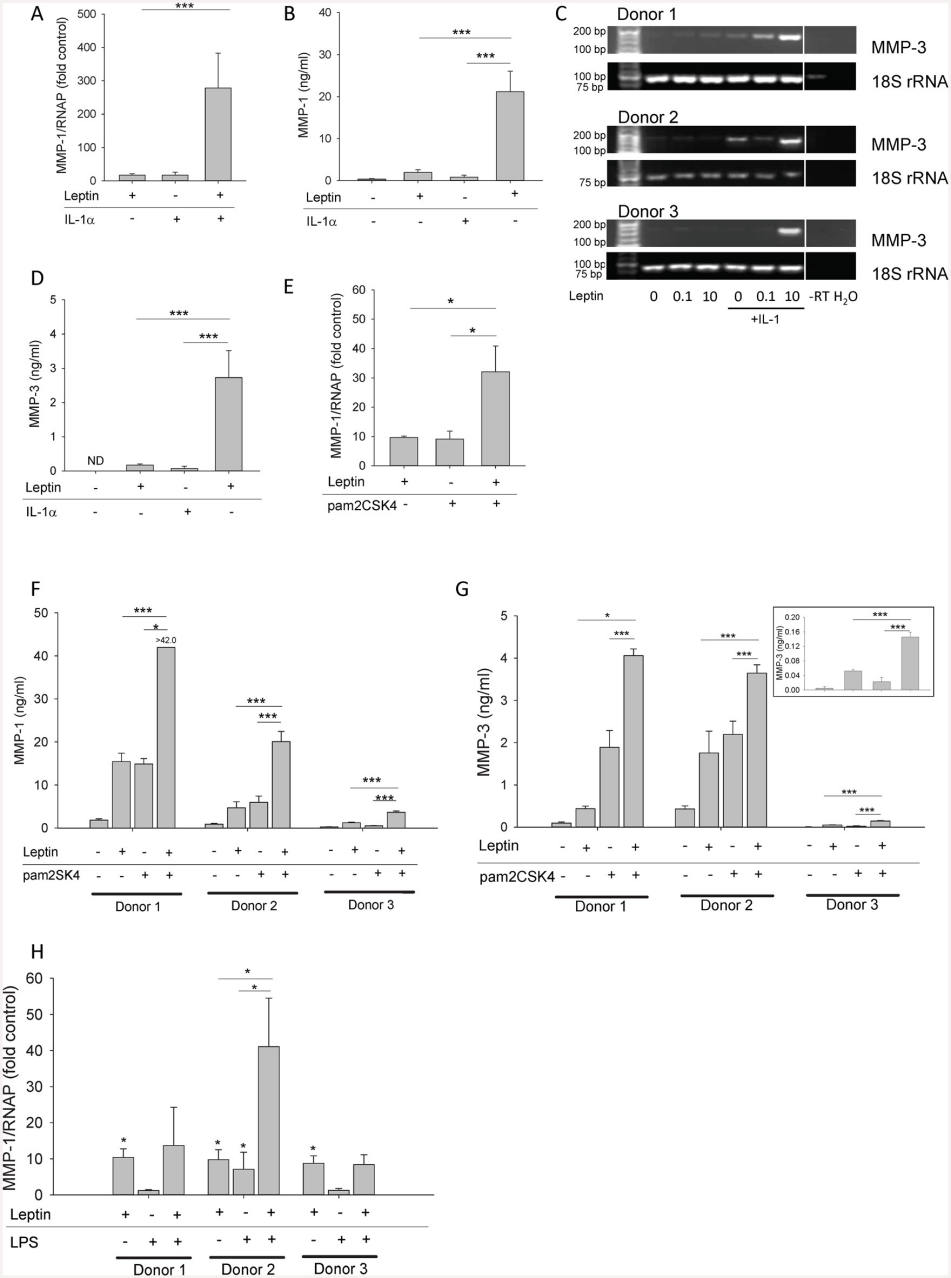
Effect of leptin and IL-1, pam2CSK4 or LPS on MMP-1 and MMP-3 production by HGFs. HGFs were stimulated with leptin (10 μg/ml), IL-1α (0.05 ng/ml) or leptin+IL-1α for 24 h (A-D). HGFs were stimulated with leptin (10 μg/ml), pam2CSK4 (50 ng/ml), or leptin+pam2CSK4 for 24 h (E-G). Inset (G): MMP-3 concentrations in HGFs isolated from donor 3 presented using an amplified scale. HGFs were stimulated with leptin (10 μg/ml), LPS (10 ng/ml) or leptin+LPS for 24 h (H). *MMP-1* gene expression was assessed by real-time RT-PCR and data (fold control) are expressed relative to RNAP with the exception of Fig 4C which illustrates MMP-3 and 18S rRNA gene expression as assessed by RT-PCR and visualized on agarose gels. Supernatant MMP-1 concentrations were assessed by ELISA. Data (A, B, D, E) are presented as mean+SEM from 3 HGF donors stimulated in independent experiments (n = 4 from each donor). Remaining data (F, G, H) are expressed as mean+SD from 3 individual HGF donors (n = 4 from each donor). *P<0.05, ***P<0.001 compared to the unstimulated control at the same time point.

**Fig 5:**
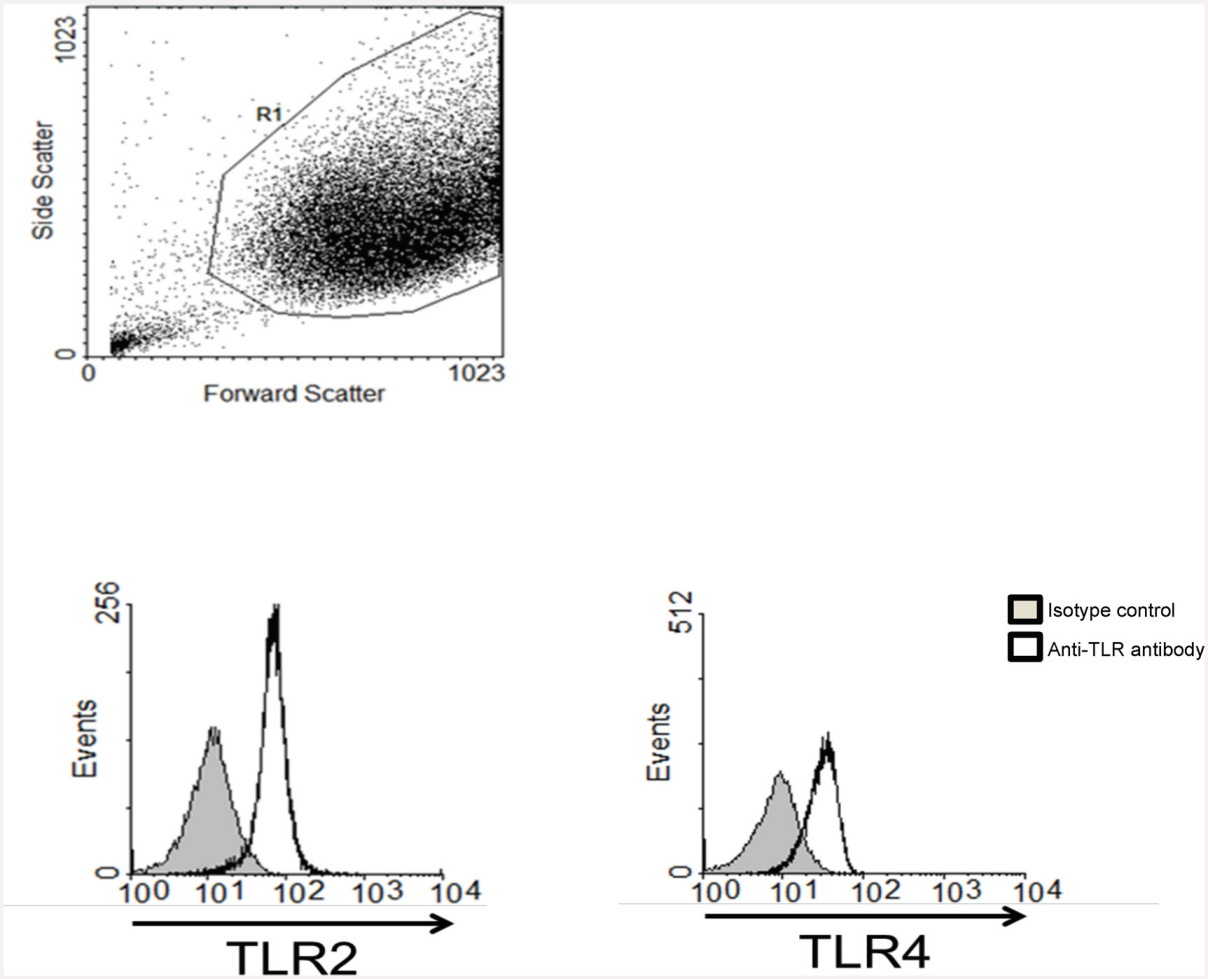
Surface TLR expression on HGFs. HGFs were prepared for flow cytometry. (A) The region highlighted in this dot plot was used to gate cells and omit cellular debris. (B) Unstimulated HGFs were analysed for cell surface TLR2 and TLR4 expression by flow cytometry. These histograms are representative of results from 4 different HGF donors.

Previous studies have demonstrated that IL-1 regulates *MMP-1/3* expression in HGFs [24,25], and that IL-1 and OSM synergistically increase MMP-1 secretion by HGFs [6]. Since we found that IL-1+OSM synergistically increased *MMP-1* mRNA levels, and MMP-1 and MMP-3 protein secretion by HGFs (Fig 3), we stimulated HGFs with leptin+IL-1. We observed a synergistic increase in *MMP-1* and *MMP-3* mRNA levels and protein secretion in HGFs stimulated with leptin+IL-1 (Fig 4A–4D). Leptin+IL-1 did not synergistically regulate the gene expression levels of membrane-type *MMP-14* (Fig 2F), *MMP-2/MMP-9*, *MMP-7* or *TIMP-1* in HGFs (not shown). Next, we stimulated HGFs with leptin in the absence or presence of pam2CSK4 or LPS, to assess synergy between leptin and these TLR agonists. Both TLR2 and TLR4 were expressed on the surface of HGFs (Fig 5). Leptin+pam2CSK4 significantly increased the expression of *MMP-1* gene and protein (Fig 4E and 4F respectively) and MMP-3 (Fig 4G) compared to HGFs stimulated with leptin or pam2CSK4 alone. However, the absolute levels of secreted MMP-1 and MMP-3 by leptin+pam2CSK4-stimulated HGFs were donor-dependent, ranging between 3.65±0.32 − >42.0 ng/ml and 0.15±0.01–4.06 ±0.16 ng/ml respectively (Fig 4F and 4G). Interestingly, synergy between leptin and LPS for *MMP-1* expression in HGFs was also observed but again appeared to be donor-dependent only in one of three donors tested (Fig 4H).

### Leptin activates MAPK, STAT and Akt signalling in HGFs

**Fig 6:**
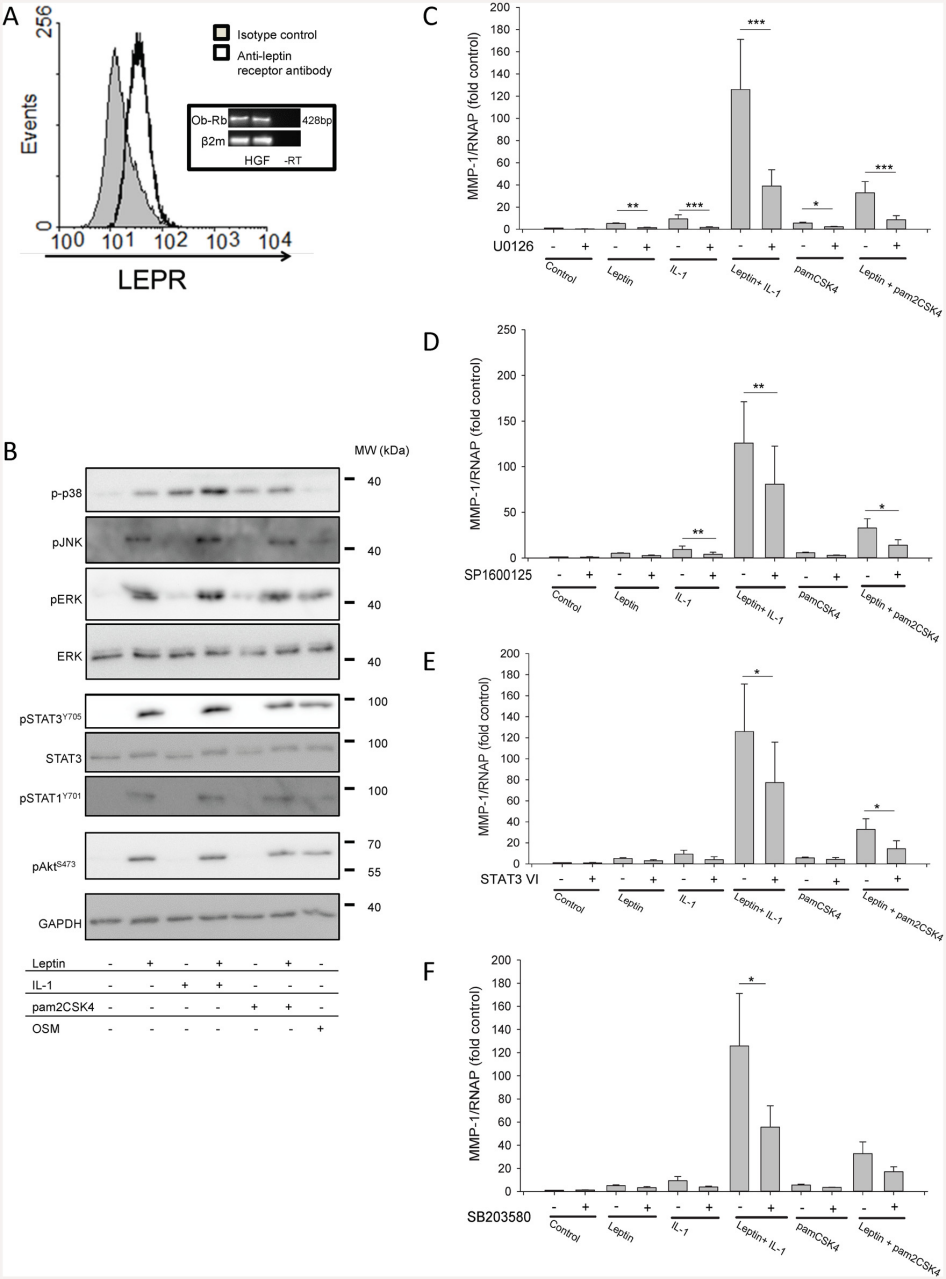
Investigation of leptin receptor expression and signalling pathways leading to MMP-1 synthesis in HGFs. Flow cytometry for cell surface leptin receptor expression on unstimulated HGFs (A). Data are representative of similar data from 4 HGF donors. Inset (A): long LEPR isoform (Ob-Rb) and β2m gene expression as assessed by RT-PCR in unstimulated HGFs. Data are representative of similar results from 7 HGF donors. HGFs were stimulated with leptin (10 μg/ml), IL-1 (0.05 ng/ml), pam2CSK4 (50 ng/ml), OSM (5 ng/ml), leptin+IL-1 or leptin+pam2CSK4 for 20 min and lysates immunoblotted with the indicated antibodies including GAPDH as a loading control (B). The blots presented are all derived from the same donor and are representative of similar results from 3 donors stimulated in independent experiments. (C) HGFs were pre-treated with the ERK inhibitor U0126 (7.5 μM), (D) the JNK inhibitor SP600126 (10 μM), (E) STAT3 inhibitor VI (100 μM), or (F) the p38 inhibitor SB203580 (10 μM) for 30 min and then stimulated with leptin (10 μg/ml), IL-1α (0.05 ng/ml), pam2CSK4 (50 ng/ml), leptin+IL-1α or leptin+pam2CSK4 for 24 h. *MMP-1* gene expression was assessed by real-time RT-PCR. Data (fold unstimulated DMSO-pre-treated control) are expressed relative to RNAP. Data are presented as mean+SEM of three donors stimulated in independent experiments (n = 4 for each donor). *P<0.05, **P<0.01, ***P<0.001 compared to the unstimulated control at the same time point.

HGFs express cell surface LEPR and the long isoform of the LEPR (Fig 6A). Stimulation of HGFs with leptin, alone or in combination with IL-1 or pam2CSK4, activated multiple signalling pathways (Fig 6B). Leptin activated the MAPK pathway in HGFs, as evidenced by phosphorylation of p38, JNK and ERK (Fig 6B). Leptin and OSM, but not IL-1 or pam2CSK4, induced STAT1 and STAT3 tyrosine phosphorylation in HGFs. Similarly, phosphorylation of Akt (at S473) in HGFs was induced by leptin and OSM, but not IL-1 or pam2CSK4 (Fig 6B).

### Multiple signaling pathways control leptin+IL-1-induced MMP-1 expression

To investigate whether these identified signaling pathways regulated the expression of leptin-induced MMP-1, HGFs were cultured in the presence of selective pathway inhibitors. The ERK inhibitor U0126 significantly reduced *MMP-1* expression in HGFs stimulated with leptin, IL-1 and pam2CSK4 both alone and in combination (Fig 6C). The JNK inhibitor SP600125 and STAT3 inhibitor VI significantly reduced *MMP-1* expression in HGFs stimulated with leptin+IL-1 or leptin+pam2CSK4 (Fig 6D and 6E), whilst the p38 inhibitor SB203580 only significantly reduced *MMP-1* expression in HGFs stimulated with leptin+IL-1 (Fig 6F). Akt inhibitor VIII at a concentration of 0.3 μM failed to inhibit *MMP-1* gene expression in HGFs stimulated with leptin, IL-1 and/or pam2CSK4 for 24 h (not shown). Thus, multiple signaling pathways are involved in mediating leptin-induced *MMP-1* expression in HGFs.

### Leptin+IL-1 alter the HGF transcriptome towards an ECM-degrading phenotype

In order to assess the extent of genes involved in ECM remodelling altered by leptin+IL-1 in HGFs, we conducted genome-wide transcriptional profiling by microarray. Numerous genes relevant to ECM remodelling were significantly regulated in HGFs by leptin, IL-1 and leptin+IL-1 compared to unstimulated HGFs (Table 4). Among these genes, the most upregulated by leptin+IL-1 were *MMP3* and *MMP1*, whilst several collagen genes (e.g. *COL6A3*, *COL14A1*, *COL15A1*) were down-regulated by leptin+IL-1 compared to the unstimulated control. Indeed, most of the genes related to ECM remodelling were only significantly regulated in HGFs stimulated with leptin+IL-1 together, rather than these mediators alone, suggesting a synergistic regulation. We found that several GO terms related to ECM remodelling were significantly overrepresented in the differentially expressed gene list for leptin+IL-1-stimulated HGFs compared to unstimulated controls (Table 5).

**Table 4:** Genes functionally related to ECM homeostasis and proteolysis that were differentially expressed in HGFs treated with leptin and/or IL-1.

Type	Symbol	Gene name	Fold change (leptin+IL-1)	Fold change (leptin)	Fold change (IL-1)
MMPs	***MMP1***	matrix metallopeptidase 1	13.0	-	4.3
	***MMP2***	matrix metallopeptidase 2	2.4	-	-
	***MMP3***	matrix metallopeptidase 3	55.9	-	12.1
	***MMP8***	matrix metallopeptidase 8	2.5	-	-
	***MMP12***	matrix metallopeptidase 12	2.0	-	-
	***MMP14***	matrix metallopeptidase 14	2.0	-	-
Other metallopeptidases	***ADAM19***	ADAM metallopeptidase domain 19	-3.0	-2.1	-2.6
	***ADAMTS1***	ADAM metallopeptidase with thrombospondin type 1 motif, 1	-	2.2	-
	***MME***	membrane metallo-endopeptidase	3.3	-	2.3
Collagens	***COL14A1***	collagen, type XIV, alpha 1	-2.2	-	-
	***COL15A1***	collagen, type XV, alpha 1	-2.1	-	-
	***COL5A1***	collagen, type V, alpha 1	-2.0	-	-
	***COL6A3***	collagen, type VI, alpha 3	-2.8	-	-
	***COL8A1***	collagen, type VIII, alpha 1	-	-	2.7
	***COL8A2***	collagen, type VIII, alpha 2	-2.0	-	-
Serine proteases	***DPP4***	dipeptidyl-peptidase 4	2.1	-	-
	***PLAT***	plasminogen activator, tissue	2.6	-	-
	***PRSS12***	protease, serine, 12	-2.4	-	-
	***PRSS23***	protease, serine, 23	-6.0	-	-2.4
Endopeptidase inhibitors	***PI3***	peptidase inhibitor 3, skin-derived	8.5	-	2.4
	***SLPI***	secretory leukocyte peptidase inhibitor	2.5	-	-
	***TIMP3***	TIMP metallopeptidase inhibitor 3	-2.3	-	-
Serine protease inhibitors	***KAZALD1***	Kazal-type serine peptidase inhibitor domain 1	-2.3	-	-
	***SERPINA3***	serpin peptidase inhibitor, clade A, member 3	2.3	-	-
	***SERPINB2***	serpin peptidase inhibitor, clade B, member 1	2.1	-	-
	***SERPINB4***	serpin peptidase inhibitor, clade B, member 4	3.3	-	-
	***SERPINB7***	serpin peptidase inhibitor, clade B, member 7	2.0	-	-
	***SERPING1***	serpin peptidase inhibitor, clade G, member 1	-	2.0	-
	***SERPINI1***	serpin peptidase inhibitor, clade I, member 1	-2.3	-	-
Glycoproteins localised to ECM	***EFEMP1***	EGF containing fibulin-like extracellular matrix protein 1	-	2.1	-
	***THBS1***	thrombospondin 1	3.3	-	-
	***THBS4***	thrombospondin 4	-2.0	-	-
	***TNC***	tenascin C	4.3	-	3.8
Proteins localised to ECM	***LAMA5***	laminin, alpha 5	-2.0	-	-
	***LAMB3***	laminin, beta 3	3.1	-	-
	***MATN2***	matrilin 2	-2.2	-	-
	***MGP***	matrix Gla protein	-4.0	-	-2.9
	***NTN4***	netrin 4	-2.2	-	-
	***OGN***	osteoglycin	-2.3		
	***POSTN***	periostin	-2.7	-	-2.1
Other ECM components or regulators of ECM components	***CSPG4***	chondroitin sulfate proteoglycan 4	-2.0	-	-
	***HAS3***	hyaluronan synthase 3	2.3	-	-
	***PLOD2***	procollagen-lysine, 2-oxoglutarate 5-dioxygenase 2	2.8	-	-
	***VCAN***	versican	-2.8	-	-2.4
Other peptidases or regulators of proteolysis	***CASP3***	caspase 3, apoptosis-related cysteine peptidase	2.9	-	-
	***CTSL***	cathepsin L	3.3	3.6	-
	***LAP3***	leucine aminopeptidase 3	2.9	-	-
	***PCOLCE2***	procollagen C-endopeptidase enhancer 2	-2.5	-	-
	***PLAUR***	plasminogen activator, urokinase receptor	2.7	-	-

**Table 5:** GO terms relevant to ECM homeostasis and proteolysis are significantly over-represented in HGFs stimulated with leptin and IL-1.

GO term		Adjusted P value	Example genes up	Example genes down
GO:0009611	response to wounding	7.66E-18	*PLAT*, *TNC*, *CASP3*, *SERPINB2*	
GO:0030198	extracellular matrix organization	5.72E-10	*MMP3*, *MMP1*, *THBS1*, *LAMB3*, *PLOD2*, *MMP8*, *HAS3*, *MMP14*, *MMP12*	*POSTN*, *NTN4*, *KAZALD1*, *LAMA5*
GO:0043062	extracellular structure organization	5.72E-10	*MMP3*, *MMP1*, *THBS1*, *LAMB3*, *PLOD2*, *MMP8*, *HAS3*, *MMP14*, *MMP12*	*POSTN*, *NTN4*, *KAZALD1*, *LAMA5*
GO:0022617	extracellular matrix disassembly	3.76E-05	*MMP3*, *MMP1*, *LAMB3*, *MMP8*, *MMP14*	*LAMA5*
GO:0032963	collagen metabolic process	3.62E-05	*MMP3*, *MMP1*, *MMP8*, *MMP2*, *MMP14*, *MMP12*	
GO:0030574	collagen catabolic process	0.000725		
GO:0052547	regulation of peptidase activity	0.00176	*PI3*, *SERPINB4*, *SLPI*, *SERPINA3*, *SERPINB2*	*TIMP3*, *COL6A3*, *PCOLCE2*, *SERPINI1*

HGFs from three donors were stimulated with leptin (10 μg/ml), IL-1β (0.05 ng/ml) or leptin+IL-1β for 24 h in independent experiments. RNA was extracted and used for genome-wide expression analysis (Illumina microarray). Genes displayed were significantly differentially expressed after leptin, IL-1 or leptin+IL-1 stimulation compared to unstimulated HGFs. The numbers displayed indicate fold change in stimulated HGFs as compared to unstimulated HGFs.—Indicates no change in gene expression.

These GO terms and example gene were significantly overrepresented in leptin+IL-1-stimulated HGFs compared to unstimulated HGFs.

**Fig 7:**
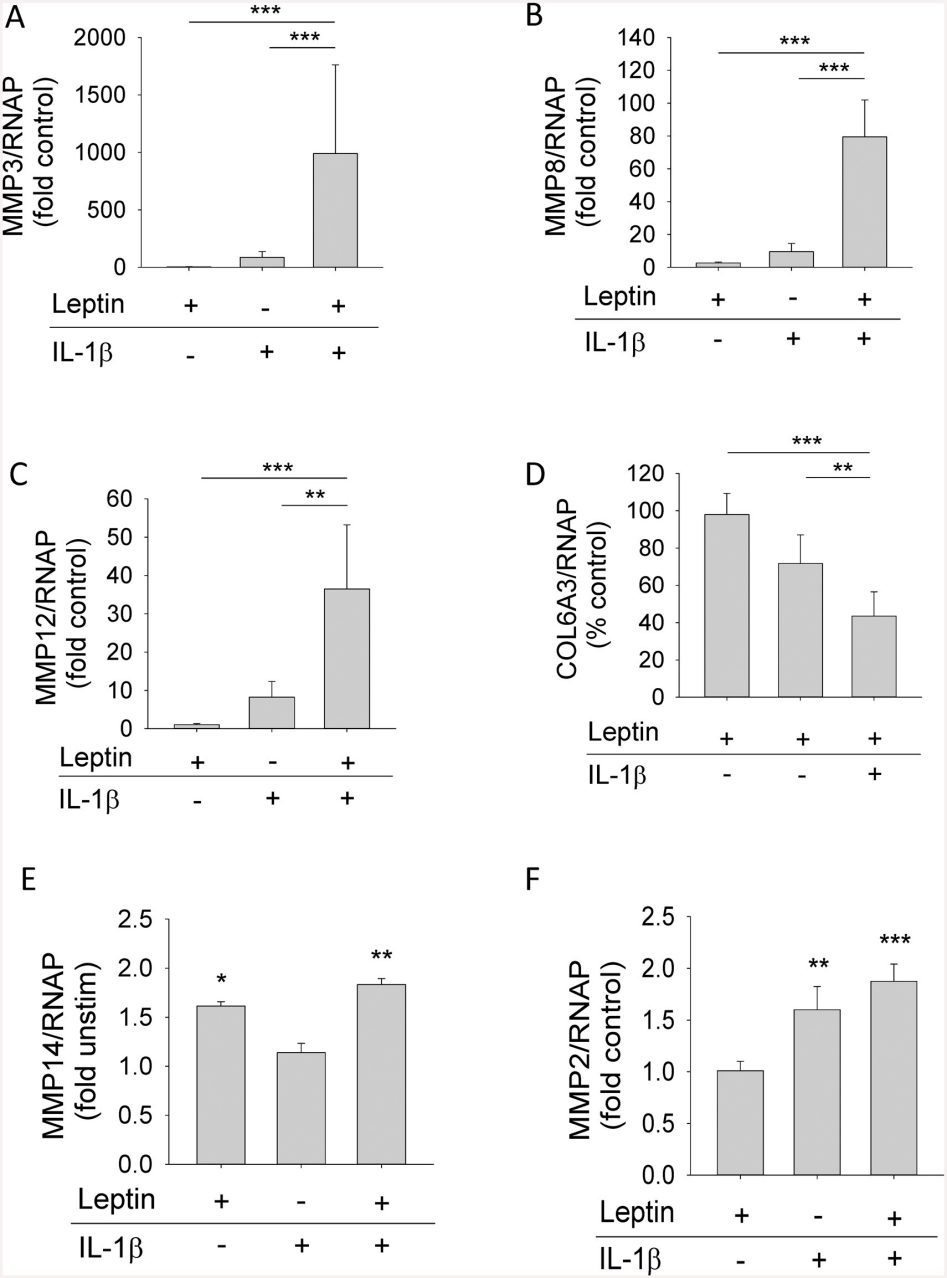
Confirmation of the microarray dataset by real-time RT-PCR. HGFs were stimulated with leptin (10 μg/ml), IL-1β (0.05 ng/ml) or leptin+IL-1β for 24 h. Real-time RT-PCR was used to assess (A) *MMP-3*, (B) *MMP-8*, (C) *MMP-12*, (D) collagen 6A3 (*COL6A3*), (E) *MMP-14*, and (F) *MMP-2* gene expression. Data are expressed relative to RNAP and are presented as mean+SEM from three donors stimulated in independent experiments (n = 4 for each donor). *P<0.05, **P<0.01, ***P<0.001 compared to the unstimulated control at the same time point.

The effect of leptin and IL-1 on the expression of several genes highlighted by transcriptional profiling (MMPs and *COL6A3*) was confirmed by real-time RT-PCR (Fig 7). Thus, leptin and IL-1 synergistically increased the expression of *MMP-3*, *MMP-8* and *MMP-12* in HGFs (Fig 7A–7C). We also confirmed that the expression of *COL6A3* was significantly lower in HGFs stimulated with leptin+IL-1 compared to either leptin or IL-1 alone (Fig 7D). Finally, we confirmed that leptin+IL-1 enhanced *MMP-14* and *MMP-2* expression in HGFs compared to unstimulated cells (Fig 7E and 7F). However, increases in *MMP-14* and *MMP-2* expression in HGFs were not due to synergy between leptin and IL-1; MMP-14 expression was stimulated by leptin independently of IL-1 (as observed in Fig 2), whilst IL-1 stimulated *MMP-2* expression independently of leptin. Although expression of leptin itself by HGFs was detected in the microarrays this was only detected at very low levels and was not influenced by any of the experimental treatments. Similarly, there was no evidence from the microarray experiments for any effect of leptin in IL-1 expression.

## Discussion

HGF-mediated ECM remodelling is implicated in the pathogenesis of periodontitis [1], but the role of adipokines in regulating this effect is not fully understood. Leptin upregulates the secretion of IL-6 and IL-8 by HGFs [15] and in the current study we demonstrated for the first time that leptin upregulates MMP-1 and MMP-3 secretion in HGFs, alone and synergistically in combination with IL-1 or pam2CSK4.

Leptin enhances the expression of MMPs in chondrocytes/cartilage [18,26] however, there are no studies concerning leptin-stimulated MMP/TIMP expression in HGFs, or indeed in any human fibroblasts. We demonstrated that the expression of certain MMPs (MMP-1, MMP-3, MMP-8, MMP-14) with different substrate preferences and extracellular locations [3] were upregulated by leptin in HGFs, which indicates that leptin stimulation of HGFs may result in the remodelling of a broad range of ECM components (including fibrillar collagens). While others have found *TIMP-1* mRNA and protein expression in HGFs is stimulated by LPS [5,27], we found no differences in *TIMP* gene expression in HGFs stimulated by leptin, IL-1, OSM, LPS or pam2CSK4 suggesting that leptin could promote ECM remodelling by HGFs by increasing the active MMP:TIMP ratio.

In agreement with previous work investigating cytokine synthesis in HGFs [28,29] we observed that MMP responses to both LPS and pam2CSK4, and leptin-stimulated MMP-3 responses were qualitatively but not quantitatively similar between HGFs from different donors. These differences might be explained by genetic and/or epigenetic differences between individuals although this requires systematic investigation.

We demonstrated that leptin and IL-1 synergistically increase the expression of multiple MMPs (MMP-1, MMP-3, MMP-8, MMP-12) in HGFs. The fold change in *MMP-1* expression in leptin+IL-1-stimulated HGFs in this study was approximately 10 times higher than that observed in a previous study in chondrocytes using the same concentrations of leptin and IL-1 [18]. Also, to our knowledge, this is the first report of synergy between leptin and a TLR2 agonist (pam2CSK4) in any primary human cells. These results indicate that the potential for HGF-mediated ECM remodelling may be greatly enhanced under simultaneous hyperleptinaemic and proinflammatory conditions, which could disturb ECM homeostasis and promote deleterious ECM degradation as is observed in periodontitis. Furthermore, leptin increases MMP-14 protein production and surface expression in primary rat cardiac fibroblasts [30]; similarly, leptin (and OSM) increased MMP-14 expression in HGFs in this study suggesting a role for IL-6 family cytokines in regulating pericellular ECM remodelling by HGFs.

All the short isoforms of the LEPR are expressed at the mRNA level by HGFs [15], including the long LEPR isoform in accordance with the data presented herein. Leptin activates the MAPK, JAK/STAT, and PI3K/Akt signalling pathways in HGFs; these are some of the well characterised signalling responses activated downstream of the long LEPR isoform [31]. Together with the finding that HGFs express cell surface LEPR, these data suggest that HGFs are sensitive and responsive to leptin.

ERK is known to regulate MMP-1 expression in IL-1-stimulated HGFs [32,33], and in leptin-stimulated chondrocytes/cartilage [18,26]. We showed that ERK regulates MMP-1 expression in HGFs stimulated with leptin, IL-1 and pam2CSK4 alone and in combination. Similarly, MAPK and STAT signalling pathways clearly regulate MMP-1 expression in HGFs stimulated by leptin+IL-1 as has been shown in chondrocytes [18]. We also showed that MMP-1 expression stimulated by leptin and pam2CSK4 is regulated by MAPK and STAT signalling. Multiple mechanisms, such as direct binding to the *MMP-1* promoter [34,35], crosstalk between signalling pathways [36], and chromatin remodelling [37] could underpin the requirement for STAT3 in regulating MMP-1 expression in HGFs. Additionally, functional redundancy between STAT family members could add another dimension to this mechanism [38]. Interestingly, Akt inhibitor VIII did not inhibit leptin (+IL-1/pam2CSK4)-induced MMP-1 expression by HGFs, which could be attributed to a requirement to use lower concentrations of this inhibitor than other studies [18,39] due to a cytotoxic effect in HGFs (not shown). Together, our results suggest that leptin and pro-inflammatory mediators stimulate specific and coordinated gingival fibroblast signalling events that determine the production of the collagenase MMP-1.

Transcriptional profiling revealed that *MMP-8* and *MMP-12* were most upregulated in HGFs after stimulation with both leptin and IL-1 together. While MMP-8 in the periodontium is predominantly thought to be neutrophil-derived [40,41], there is evidence that other cells in the periodontium such as plasma cells, ‘macrophage-like cells’, sulcular epthielial cells as well as HGFs synthesise MMP-8 [25,41]. The elastase MMP-12 can activate itself and other MMPs through autolytic processing [42], providing a putative mechanism by which the initial activation of HGF-derived MMPs upregulated by leptin+IL-1 could occur. Interestingly, leptin+IL-1, as compared to leptin or IL-1 alone, synergistically regulated a wide range of genes relevant to ECM remodelling not limited to MMPs including collagens and other enzymes as well as certain cytokines and chemokines (unpublished observations). Studies that investigate the full extent of the HGF phenotype induced by leptin and the functional implications of this, in relation to ECM remodelling and to broader inflammatory responses in the gingiva are clearly required, and may help to address the possible redundancy between leptin and other IL-6 family members (e.g. OSM).

Circulating leptin concentrations are proportional to adipose tissue mass, and as such leptin has been shown to indicate organismal energy status to several tissues/organs [11] and also influence the immunocompetence of leukocytes [43]. We hypothesise that leptin could serve to inform HGFs of the energy status of an organism, thereby ensuring that the responses of HGFs (e.g. ECM remodelling) are in line with energy availability but are influenced by inflammatory context. For example, the presence of high concentrations of leptin in the inflamed gingiva (and hence a pro-inflammatory cytokine milieu) may synergistically drive HGF-mediated ECM degradation.

We conclude that leptin, in synergy with pro-inflammatory stimuli (IL-1 and the TLR-2 agonist pam2CSK4) selectively enhances MMP-1 and MMP-3 secretion in HGFs and suggest that gingival fibroblast-mediated ECM degradation, may be deleteriously enhanced during conditions of hyperleptinaemia (e.g. obesity, type 2 diabetes mellitus, exogenous leptin therapy).
